# Mediating role of emotional labor in the association between emotional intelligence and fatigue among Chinese doctors: a cross-sectional study

**DOI:** 10.1186/s12889-018-5817-7

**Published:** 2018-07-16

**Authors:** Li Liu, Peiyao Xu, Kexin Zhou, Jiayu Xue, Hui Wu

**Affiliations:** Department of Social Medicine, School of Public Health, China Medical University, No.77 Puhe Road, Shenyang North New Area, Shenyang, Liaoning 110122 People’s Republic of China

**Keywords:** Fatigue, Emotional intelligence, Emotional labor, Moderating role, Doctors, Hospital management

## Abstract

**Background:**

Fatigue is highly prevalent among doctors worldwide. However, no research has been done to examine the associations of emotional intelligence (EI) and emotional labor strategy with fatigue among Chinese doctors. This study aimed to examine whether or not emotional labor strategy mediates the association between EI and fatigue in this occupational group.

**Methods:**

A cross-sectional study was conducted in Shenyang from March to April 2014. A set of self-administered questionnaires was distributed to 950 doctors, including Chalder Fatigue Scale (CFS), Wong and Law Emotional Intelligence Scale (WLEIS) and a 14-item emotional labor scale. Complete responses were obtained from 740 (77.9%) participants. Hierarchical linear regression was performed to examine the associations of EI and emotional labor strategies (surface acting, SA; deep acting, DA; natural acting, NA) with fatigue. Asymptotic and resampling strategies were used to examine the mediating roles of emotional labor strategies.

**Results:**

The mean score of fatigue was 8.02 (SD = 3.39). After adjusting for age, gender, marital status, job rank, monthly income, weekly working time, shift and department, EI was negatively associated with fatigue (*β* = − 0.270, *P* <  0.001). SA was positively associated with fatigue (*β* = 0.168, *P* <  0.001), whereas NA was negatively associated with fatigue (*β* = − 0.105, *P* = 0.004); however, DA was not significantly associated with fatigue (*β* = 0.034, *P* = 0.381). Thus, SA (a × b = − 0.026, BCa 95% CI: − 0.050, − 0.011) and NA (a × b = − 0.024, BCa 95% CI: − 0.046, − 0.006) significantly mediated the association between EI and fatigue, respectively.

**Conclusions:**

There was a high level of fatigue among Chinese doctors. EI could indirectly reduce fatigue partially through modifying SA and NA strategies, respectively. EI intervention, education and training in emotional labor should be carried out to cope with fatigue.

## Background

Fatigue in the workplace comprises both physical and mental constructs, affecting the overall state of workers. Reduced proprioception and strength due to fatigue may lead to low work performance and quality, and high incidence of human errors and accidents [1, 2]. Moreover, fatigue may also result in many adverse health outcomes such as chronic fatigue syndrome, burnout, and work-related musculoskeletal disorders in the long term [3, 4]. Among the groups of healthcare occupations, fatigue has been described as a serious problem, especially doctors [5–8]. Fatigue is highly prevalent among doctors in China [7, 8]. Due to the shortage of health care professionals, doctors often work overload, and they have to work in an environment with various stressors, which make them prone to suffering from fatigue [6, 9]. It could not only affect the physical health of doctors, but also lead to decline in work performance and increase of mental health problems [7, 8, 10]. At the same time, the decline of work quality and efficiency caused by fatigue could directly impair patients’ satisfaction with health care services and the doctor-patient relationship. These stressors in turn will aggravate physical and mental fatigues, thus forming a vicious circle [11]. Therefore, great attention should be paid to the etiology of fatigue in order to develop appropriate measures to prevent it among doctors.

In workplaces, fatigue may be a consequence of highly demanding work and various psychological and environmental factors. In doctors, in addition to physical fatigue caused by high workload, it is worth paying more attention to mental fatigue caused by adverse occupational psychological environment. Work stress perceived by doctors leads to negative emotional reactions and gradually consumes their energy. What’s more, mental fatigue can facilitate the perception of physical fatigue [12]. Therefore, effective emotion regulation may have an important preventive role on fatigue [13]. Emotional intelligence (EI), its concept was first proposed by Salovey and Mayer in 1990 [14], consisting of the abilities to identify, understand, harness and regulate emotions in oneself and in others. In the processes of diagnosis and treatment, doctors need to show specific and appropriate emotions to their patients in a high-stress environment. Also, doctors have to interact frequently with other people, such as patients’ family members, nurses, and consultants from other departments. Obviously, for doctors, EI is an important internal resource for dealing with interpersonal emotional pressure in those processes. For instance, doctors with higher EI are more likely to relieve their work stress and reduce tiredness by control emotions [15, 16]. A previous research has reported that the doctors who used the practical skills of EI, such as confidence, empathy, adaptability and conflict management, to communicate successfully with patients and colleagues might feel satisfied with their works [16].

Emotional labor is the process of managing feelings and expressions to achieve the professional requirements of emotion when interacting with customers, co-workers and superiors [17]. According to the results of Diefendorff’s research, the performance of emotional labor strategy mainly includes three aspects: surface acting (SA, modifying facial expressions), deep acting (DA, modifying inner feelings) and natural acting (NA). Roles that have been identified as requiring emotional labor include many occupations in the tertiary/service sector [18], particularly within healthcare settings. Therefore, there is a great deal of emotional labor for health care workers who need face-to-face contact with patients for a long time at work [19, 20]. Hu et al. reported that the most frequently adopted strategy by doctors was NA, followed by DA and SA [20]. In addition, as previously mentioned, SA could be more consistently problematic for employees’ well-being than DA. When employees play SA, their self-authenticities are damaged because of the internal and external conflict in emotion, and they will be prone to psychological distress, fatigue and job dissatisfaction [21–23]. Conversely, DA is an effortful process through which employees change their internal feelings to align with organizational expectations, and it is positively associated with organizational behavioral outcomes and physical and mental well-being. Therefore, health care providers should continuously prioritize and integrate these complex emotions and specific situations.

According to the results of many previous studies, EI has various effects on emotional labor strategies [23–25]. In some extent, EI can reduce SA, and improve DA and NA. Specifically, the doctor who has high EI could easily understand the emotions of patients in communication with them. In general, they don’t need to pay efforts to change their emotions, and natural emotion outpouring can satisfy organizational expectations. However, when internal feelings are not aligned with organizational expectations, doctors with high EI have adequate abilities to arouse or suppress their emotions [26]. Therefore, EI could play its positive roles by influencing emotional labor strategies. Moreover, EI and emotional labor strategies can be changed through effective intervention to enable them to cope with stressful environments and prevent physical and mental fatigues in workplaces. However, no research has been done to examine the associations of EI and emotional labor strategies with fatigue among Chinese doctors to our best knowledge.

In light of the above concerns, the purpose of the present study was to verify the following three assumptions among Chinese doctors: 1) EI and emotional labor strategies are associated with fatigue, 2) EI is associated with emotional labor strategies, and 3) emotional labor strategies mediate the association between EI and fatigue.

## Methods

### Study design and sample

A cross-sectional study was conducted in Shenyang, the capital city of Liaoning Province, from March to April 2014. The city comprises thirteen districts, and five districts were selected in this study. From each selected district, two general hospitals were randomly selected. Thus, a total of ten large general hospitals were included in the present study. In China, a secondary hospital should possess 101–500 beds, while a tertiary hospital is required to have more than 500 beds. This study included six secondary hospitals and four tertiary hospitals. After obtaining a written informed consent from each participant, a set of self-administered questionnaires was directly distributed to 950 doctors from different departments. These questionnaires were completed anonymously in a private room after the respondent’s shift. Complete responses were obtained from 740 participants (effective response rate: 77.9%). The study was conducted in accordance with the Declaration of Helsinki, and the protocol was approved by the Committee on Human Experimentation of China Medical University. In this study, all of the participants were voluntary and anonymous, and written informed consent was provided by them.

### Measures

#### Measurement of fatigue

The Chalder Fatigue Scale (CFS) was used to measure fatigue severity [27]. This is a 14-item dichotomized survey with two components, physical fatigue (8 items) and mental fatigue (6 items). Each item describes a fatigue-related problem with two responses, 0 (no fatigue-related problem) and 1 (have fatigue-related problem). A total fatigue score was calculated ranging from 0 to 14 in the study. A higher CFS-14 score indicates a higher fatigue severity. The Chinese version of the CFS-14 has been used in various occupational groups in China, and it has adequate reliability and validity [7, 8, 28]. In the present study, the Cronbach’s alpha coefficient of the total CFS-14 was 0.79.

#### Measurement of EI

The Wong and Law Emotional Intelligence Scale was used to measure EI [29]. The WLEIS is comprised of 16 items with four subscales corresponding to the four components of EI: appraisal of self-emotions, appraisal of others’ emotions, use of emotion on cognition, and regulation of emotion. Each item in the WLEIS uses a 7-point Likert scale ranging from 1 (totally disagree) to 7 (totally agree). A total EI score was calculated ranging from 16 to 112 due to our interest in the overall construct. A higher score indicates a higher EI. The Chinese version of the WLEIS has shown adequate internal consistency and validity [15, 30]. In this study, the Cronbach’s alpha for the total WLEIS was 0.95.

#### Measurement of emotional labor

The 14-item scale described by Dieffendorff et al. (2005) was used to assess emotional labor [17]. The scale items cover SA (7 items), DA (4 items), and NA (3 items). Each item is scored on a 5-point Likert scale ranging from 1 (strongly agree) to 5 (strongly disagree). Thus, the summed score of the three subscales ranged from 7 to 35, 4 to 20, and 3 to 15, respectively. A higher score indicates a higher emotional labor strategy. Some rewordings were applied to take account of the current sample. For example, the word “customers” in original items was replaced by “patients”. The Chinese scale has been widely applied among Chinese occupational groups with good reliability and validity [19, 20, 31]. The Cronbach’s alpha coefficients of the SA, DA, and NA subscales were 0.88, 0.79, and 0.89 in this study, respectively.

#### Demographic characteristics

Demographic factors included gender, age (yrs), marital status and education. Marital status was categorized as single/widowed/divorced/separated and married/cohabitated. Education was categorized as junior college or lower, college, and postgraduate or higher.

#### Working characteristics

Working characteristics including job rank, monthly income (RMB, yuan/US$), weekly working time (hrs), shift, hospital type and department were collected in this study. Job rank was categorized as staff and director. Monthly income was categorized as ≤ 3000 yaun/US$461, > 3000 yuan/US$461–4000 yuan/US$615, and > 4000 yuan/US$615. Weekly working time was divided into four groups: ≤ 40 h, 41–50 h, 51–60 h, and > 60 h. The work shift was defined as yes or no. Hospital type was categorized as secondary hospital and tertiary hospital. The departments of the participants were divided into five categories: internal medicine, surgery, obstetrics and gynecology, pediatrics, and others (dermatology, rehabilitation, blood purification, traditional Chinese medicine, anesthesiology, etc.).

### Statistical analysis

The group differences of fatigue were analyzed by Student’s *t*-test or one-way ANOVA. Correlations among continuous variables were examined using Pearson’s correlation. Hierarchical linear regression analysis was performed to examine the associations of EI and emotional labor strategies (SA, DA and NA) with fatigue. EI was modeled as an independent variable, with fatigue as an outcome; SA, DA and NA as mediators, and demographic and working characteristics as covariates. In step 1, demographic and working variables were added. In step 2, EI was added. In step 3, SA, DA and NA were added. Variance inflation factor (VIF) was calculated to identify multi-collinearity. Asymptotic and resampling strategies were used to examine the three emotional labor strategies as potential mediators in the association between EI and fatigue based on 5000 bootstrap samples [32]. A bias-corrected and accelerated 95% confidence interval (BCa 95% CI) was estimated for each mediation (a × b product), and a BCa 95% CI excluding 0 indicated a significant mediating role. Statistical analysis was executed by SPSS 19.0 software and a two-tailed *P* <  0.05 was viewed as statistically significant.

## Results

### Participant characteristics

The demographic and working characteristics of subjects and comparisons on fatigue are shown in Table 1. There was a significant difference (*t* = 2.456, *P* = 0.014) in the level of fatigue between men and women. Married/cohabited doctors reported a higher fatigue score compared with those single/divorced/widowed/separated (*t* = 3.203, *P* = 0.001). However, educational level was not significantly related to the level of fatigue. In view of working characteristics, job rank, monthly income, weekly working time, shift and department were all significantly related to the score of fatigue. However, hospital type was not significantly related to the fatigue of doctors.

**Table 1:** Demographic and working characteristics of subjects and comparisons on fatigue

Variables	*n*	%	Mean	SD	*F/t*	*P*
Gender					2.456	0.014
Men	299	40.4	8.39	3.48		
Women	441	59.6	7.76	3.30		
Marital status					3.203	0.001
Single/divorced/widowed/separated	130	17.6	7.16	3.66		
Married/cohabited	610	82.4	8.20	3.30		
Educational level					1.831	0.161
Junior college or lower	77	10.4	8.53	3.50		
College	459	62.0	8.07	3.34		
Postgraduate or higher	204	27.6	7.70	3.45		
Job rank					3.188	0.001
Staff	624	84.3	8.19	3.37		
Director	116	15.7	7.10	3.36		
Monthly income (RMB, yuan/US$)					4.462	0.012
≤ 3000/461	377	50.9	8.18^a^	3.38		
> 3000/461–4000/615	226	30.5	8.21^b^	3.36		
> 4000/615	137	18.5	7.24	3.37		
Weekly working time (hrs)					11.317	< 0.001
≤ 40	62	8.4	7.54^c^	3.62		
41–50	293	39.6	7.26^d,e^	3.40		
51–60	153	20.7	8.35	3.19		
> 60	232	31.4	8.88	3.21		
Shift					2.508	0.012
Yes	438	59.2	8.27	3.31		
No	302	40.8	7.64	3.47		
Hospital type					1.352	0.177
Secondary	339	45.8	7.83	3.36		
Tertiary	401	54.2	8.17	3.41		
Department					4.642	0.001
Internal medicine	336	45.4	7.85^f^	3.41		
Surgery	198	26.8	8.77	3.21		
Obstetrics and gynecology	63	8.5	7.36^g^	3.45		
Pediatrics	73	9.9	7.13^h^	3.24		
Others	70	9.4	8.17	3.51		

*Abbreviations SD* standard deviation

^a,b^Significantly higher compared with > 4000 yuan/US$615 group, *P* <  0.01; ^c^Significantly lower compared with > 60 h group, *P* <  0.01; ^d^Significantly lower compared with > 60 h group, *P* < 0.001; ^e^Significantly lower compared with 51–60 h group, *P* < 0.001; ^f,g,h^Significantly lower compared with surgery group, *P* < 0.001

### Correlations among study variables

Correlations among study variables are presented in Table 2. The mean age of our sample was 39.20 yrs. (SD = 8.78), and the mean score of fatigue was 8.02 (SD = 3.39). Age was negatively correlated with SA and DA, respectively. EI was negatively correlated with SA and fatigue, but was positively correlated with NA. SA and DA were positively correlated with fatigue, whereas NA was negatively correlated with fatigue.

**Table 2:** Correlations among study variables

Variables	Mean	SD	1	2	3	4	5
1. Age (yrs)	39.20	8.78	1				
2. EI	81.29	14.76	−0.010	1			
3. SA	19.40	5.91	−0.079*	− 0.196**	1		
4. DA	12.72	3.12	−0.130*	−0.014	0.424**	1	
5. NA	10.60	2.33	0.010	0.232**	−0.121**	0.205**	1
6. Fatigue	8.02	3.39	0.032	−0.365**	0.278**	0.091*	−0.200**

*Abbreviations SD* standard deviation, *EI* emotional intelligence, *SA* surface acting, *DA* deep acting, *NA* natural acting

*P < 0.05; **P < 0.01

### Associations of EI and emotional labor strategies with fatigue

The results of hierarchical linear regression analysis on the associations of EI and the three emotional labor strategies with fatigue are presented in Table 3. Firstly, the VIFs of all independent variables were less than 1.6, suggesting that multi-collinearity was not an issue in the estimate. After adjusting for age, gender, marital status, job rank, monthly income, weekly working time, shift and department in step 2, EI was negatively associated with fatigue (*β* = − 0.320, *P* <  0.001). EI accounted for an additional 9.7% of the variance of fatigue. In step 3, SA was positively associated with fatigue (*β* = 0.168, *P* <  0.001), whereas NA was negatively associated with fatigue (*β* = − 0.105, *P* = 0.004); however, DA was not significantly associated with fatigue (*β* = 0.034, *P* = 0.381). These emotional labor strategies accounted for an additional 4.6% of the variance of fatigue. When these emotional labor strategies were added in step 3, the absolute value of EI’ *β* was diminished. Thus, emotional labor strategies could probably become mediators in the association between EI and fatigue.

**Table 3:** Associations of EI and emotional labor strategies with fatigue

Variables	Step 1			Step 2			Step 3		
	*β*	*P*	VIF	*β*	*P*	VIF	*β*	*P*	VIF
Age	0.071	0.113	1.574	0.052	0.222	1.577	0.066	0.108	1.594
Gender	−0.008	0.848	1.395	−0.016	0.690	1.396	−0.033	0.700	1.429
Marital status	0.098	0.015	1.294	0.088	0.022	1.296	0.087	0.019	1.297
Job rank	−0.105	0.014	1.442	−0.077	0.057	1.450	−0.077	0.050	1.452
Monthly income	−0.083	0.033	1.211	−0.074	0.047	1.212	−0.072	0.045	1.215
Weekly working time	0.164	< 0.001	1.362	0.137	0.001	1.370	0.121	0.002	1.396
Shift	0.024	0.754	1.357	−0.004	0.917	1.365	−0.019	0.617	1.370
Department									
Dummy_1	0.129	0.003	1.507	0.095	0.022	1.518	0.094	0.020	1.526
Dummy_2	0.012	0.754	1.180	0.015	0.686	1.180	0.027	0.441	1.185
Dummy_3	0.020	0.627	1.285	0.032	0.395	1.287	0.032	0.397	1.294
Dummy_4	0.064	0.095	1.152	0.061	0.094	1.152	0.073	0.038	1.158
EI				−0.320	< 0.001	1.063	−0.270	< 0.001	1.136
SA							0.168	< 0.001	1.477
DA							0.034	0.381	1.427
NA							−0.105	0.004	1.240
*F*	5.954			13.220			14.033		
Adjusted *R*^*2*^	0.069			0.166			0.209		
Δ*R*^*2*^	–			0.097			0.046		

Gender, men versus women; Marital status, married/cohabitated versus single/widowed/divorced/separated; Job rank, director versus staff; Shift, yes versus no. Department: Dummy_1, surgery versus internal medicine; Dummy_2, obstetrics and gynecology versus internal medicine; Dummy_3, pediatrics versus internal medicine; Dummy_4, others versus internal medicine. Abbreviations: *EI* emotional intelligence, *VIF* variance inflation factor, *SA* surface acting, *DA* deep acting, *NA* natural acting

### Mediating roles of emotional labor strategies

Based on the results of hierarchical linear regression analysis in Table 3, asymptotic and resampling strategies were used to examine the mediating roles of emotional labor strategies. As shown in Table 4, EI was negatively associated with SA (a = − 0.157, *P* <  0.01), whereas EI was positively associated with NA (a = 0.232, *P* <  0.01). Thus, SA (a × b = − 0.026, BCa 95% CI: − 0.050, − 0.011) and NA (a × b = − 0.024, BCa 95% CI: − 0.046, − 0.006) significantly mediated the association between EI and fatigue, respectively. In addition, the proportions of mediating roles of SA and NA were 8.1 and 7.5% in the total effect of EI on fatigue, respectively.

**Table 4:** Mediating roles of emotional labor strategies

Mediators	a	b	a × b (BCa 95% CI)
SA	−0.157**	0.169**	−0.026* (−0.050, − 0.011)
DA	−0.013	0.034	–
NA	0.232**	−0.105**	−0.024* (− 0.046, − 0.006)

*Abbreviations SA* surface acting, *DA* deep acting, *NA* natural acting, *BCa 95% CI* bias-corrected and accelerated 95% confidence interval

*P < 0.05; **P < 0.01. Age, gender, marital status, job rank, monthly income, weekly working time, shift and department were adjusted

## Discussion

The result of the present study showed that the mean score of fatigue was 8.02 ± 3.39 among Chinese doctors of Shenyang, Liaoning Province, and it was higher than the result of a previous study in the same occupational group of Hainan Province in China [7], and basically consistent with the fatigue level of doctors (8.03 ± 2.87) from Anshan of Liaoning Province [33]. Lin et al. reported a higher fatigue level among Chinese doctors of Zhuhai, Guangdong Province [8]. The fatigue level of doctors in this study was also higher than the results of general population (5.50 ± 3.09) [34] and scientific and technical personnel (7.28 ± 3.37) [28] in China. In the United States, the physical and mental fatigues of doctors are very common. O’Donnell et al. had reported that nearly half of physicians (45.4%) had high levels of fatigue, and there was a strongly positive association between fatigue and dissatisfaction with practicing medicine [5]. Also, doctors in Japan are suffering from prolonged fatigue [6, 35]. The fatigue of doctors is associated with sick leave and injury, and it can increase the frequency of task errors and affect the safety of patients. As a common and serious problem among doctors worldwide, fatigue needs to be paid more attention by themselves and hospital managers.

In this study, EI had a significantly negative association with fatigue, and this result is consistent with findings from other studies [36, 37]. The finding of Zeidner et al. indicated that self-report trait EI was inversely associated with compassion fatigue in health-care professionals [36]. Similarly, Weng et al. found that higher self-rated EI was significantly associated with less burnout among internists [15]. One possible explanation for these findings is that EI could facilitate the development of individuals’ coping resources, such as healthy emotions, adaptive explanations and adequate social support, which help them prevent physical and mental fatigues.

The three emotional labor strategies had different effects on fatigue among Chinese doctors of Shenyang in this study. Our result showed that SA was positively associated with fatigue, whereas there was a negative association between NA and fatigue. Because of the inconsistency between the use of SA and cognitive evaluation and emotional experience, doctors need to passively exert some efforts to maintain their external emotional performance. This not only consumes physiological and psychological resources, but also causes fatigue when these consumed resources can’t be effectively supplemented [38]. However, DA is an internal emotional experience, and it is adjusted through positive thinking and striving [39]. Although DA has considerable positive effects on both individual and organizational outcomes, it still involves a great expenditure of energy to deeply modify one’s feelings in the process of adjustment [40]. For NA, employees do not need to adjust their emotions in cognition, and they naturally come up with the emotions that have been experienced in the workplace. Obviously, NA is a daily, without effort, and not a source of stress. Thus, NA was negatively associated with the level of fatigue among Chinese doctors in this study.

In addition, EI was an important influencing factor of the performance of emotional labor strategy in this study [23–25], which is mainly reflected in the two strategies: SA and NA. The results indicated that EI was significantly and negatively associated with SA strategy, and positively associated with NA strategy, but did not significantly affect DA strategy [23]. Specifically, doctors with higher EI are more inclined to use NA strategy. Those doctors are naturally able to feel empathic in the face of patients’ various situations, and express the emotions in line with the organization’s requirements. In this case, they almost do not need to pay any emotional effort and cost. However, for doctors with lower EI, they often adopt SA strategy, because they can’t really understand their own feelings, control emotions and act reasonably, resulting in the inability to reconcile their internal emotions with supposed emotions. This study also firstly proved that emotional labor strategies mediated the association between EI and fatigue among Chinese doctors. Specifically, EI could lead to the reduction of SA strategy, and promote the use of NA strategy. Thus, EI directly and indirectly decreased the doctor’s perception of fatigue.

In addition, the study found that the doctors who married/cohabited are more prone to fatigue than those single/divorced/widowed/separated doctors, because the former shoulder the dual responsibilities and pressures from work and family, which aggravates fatigue [9]. There was a significantly negative association between high job rank and fatigue [9]. Although directors have to undertake both medical care and management tasks, they have greater autonomy and higher rewards at work than general practitioners. What’s more, job position is usually proportional to working income, and monthly income was negatively associated with the level of fatigue in this study [6]. Therefore, equitable work income is a protective factor in coping with fatigue. Consistent with some previous research results [6, 9], weekly working time was positively associated with the level of fatigue in this study. Therefore, it is recognized that the rationalization of working time is an effective measure. In addition, the department of doctors was related to the level of fatigue [33]. In particular, surgeons could tend to report a higher fatigue than internists. A reasonable reason is that surgeons often have to spend a lot of physical energy during their surgical operations.

According to the results of this study, fatigue could be reduced from the perspectives of EI and emotional labor strategies. First of all, hospital managers should provide doctors with the opportunities and ways to learn emotional self-regulation, in order to improve their EI levels gradually [41]. Secondly, emotional labor education and training should be carried out to avoid the formation of SA strategy [18, 42], which could reduce the physical and mental fatigues of doctors.

Before conclusions can be drawn, several limitations of this study must be acknowledged. Firstly, due to cross-sectional design, it can not to assess the causal relationships among study variables in the study, which need to be confirmed in longitudinal study. In fact, our research hypotheses were based on solid theoretical and research foundations. Secondly, the present study was only conducted at the secondary and tertiary general hospitals in a provincial capital city, in northeast China. Therefore, further research is needed to improve the generalization of our findings among doctors from hospitals in different levels and regions. Additionally, the associations among study variables might be affected by the unique use of self-report measures. Measurement tools with adequate reliability and validity and the anonymity of respondents were adopted to reduce common-method bias.

## Conclusions

There was a high level of fatigue among Chinese doctors. EI was negatively associated with fatigue. SA was positively associated with fatigue, and NA was negatively associated with fatigue. However, DA was not significantly associated with fatigue. EI could indirectly reduce fatigue partially through modifying SA and NA strategies, respectively. Therefore, EI intervention, as well as education and training in emotional labor should be carried out to cope with fatigue among Chinese doctors.

## Abbreviations

BCa 95% CI: Bias-corrected and accelerated 95% confidence interval
CFS: Chalder Fatigue Scale
DA: Deep acting
EI: Emotional intelligence
NA: Natural acting
SA: Surface acting
SD: Standard deviation
VIF: Variance inflation factor
WLEIS: Wong and Law Emotional Intelligence Scale
